# Expression of estrogen and progesterone receptors in vestibular schwannomas and their clinical significance

**DOI:** 10.1186/1477-5751-8-9

**Published:** 2009-11-04

**Authors:** Sushila Jaiswal, Vinita Agrawal, Awadhesh Kumar Jaiswal, Rakesh Pandey, Ashok Kumar Mahapatra

**Affiliations:** 1 Department of Pathology and Neurosurgery, Sanjay Gandhi Postgraduate Institute of Medical Sciences, Raebareli Road, Lucknow, 226014, India

## Abstract

**Objective:**

The objective was to determine the expression of estrogen and progesterone receptors in vestibular schwannomas as well as to determine predictive factors for estrogen and progesterone receptor positivity.

**Materials and methods:**

The study included 100 cases of vestibular schwannomas operated from January 2006 to June 2009. The clinical details were noted from the medical case files. Formaldehyde-fixed parafiin-embedded archival vestibular schwannomas specimens were used for the immunohistochemical assessment of estrogen and progesterone receptors.

**Results:**

Neither estrogen nor progesterone receptors could be detected in any of our cases by means of well known immunohistochemical method using well documented monoclonal antibodies. In the control specimens, a strongly positive reaction could be seen.

**Conclusion:**

No estrogen and progesterone receptor could be found in any of our 100 cases of vestibular schwannomas. Hence our study does not support a causative role of estrogen and progesterone in the growth of vestibular schwannoma as well as hormonal manipulation in the treatment of this tumor.

## Introduction

Estrogen and progesterone receptors have been reported in various human tumors, including endometrial carcinoma, breast carcinoma, and carcinoma of prostate. They play a crucial role in the treatment of breast carcinoma. The presence of these receptors has been examined in a number of intracranial tumors, in particular in meningiomas. It has been suggested that these hormones could be involved in the development of meningiomas and that their influence could explain the greater frequency of meningiomas in women than in men, their increased growth rate during pregnancy, and their association with breast cancer [1,2]. Schwannomas are also more frequent, larger and more vascular in women and increased growth rate during pregnancy has been described. There are several articles on the potential role for the therapeutic manipulation of estrogen and progesterone receptors in meningiomas that have not responded to other therapy [1,2].

**Figure 1:**
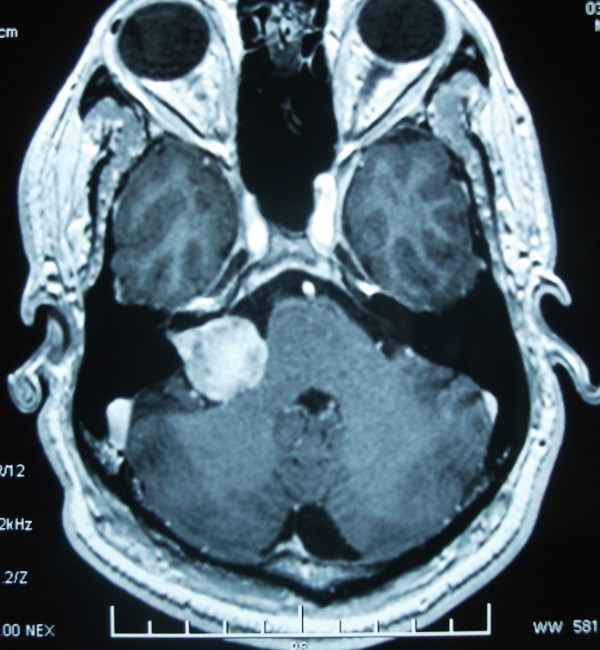
**Magnetic Resonance (MR) image of vestibular schwannoma**.

Vestibular schwannoma also known as acoustic schwannoma is the most common cerebellopontine angle tumor and represents 9% of all brain tumors (Figure 1). Expression of estrogen and progesterone receptors and their potential role in the progression of vestibular schwannoma is still an area of controversy. Many diverging studies, using various biochemical and immunohistochemical methods, have been published on the contents of estrogen and progesterone receptors in vestibular schwannomas [3-13]. These studies may help in considering endocrinological therapy for the vestibular schwannoma especially in recurrent and residual cases where complete excision is not feasible.

The objective of the present study was to retrospectively determine the expression of estrogen and progesterone receptors by means of immunohistochemical methods using monoclonal antibodies in 100 cases of vestibular schwannomas and also to determine predictive factors for estrogen and progesterone receptor positivity.

## Materials and methods

### Patient population

This retrospective study included 100 cases of vestibular schwannomas operated from January 2006 to June 2009 in the Department of Neurosurgery, Sanjay Gandhi Postgraduate Institute of Medical Sciences, Lucknow, India. The clinical parameters like age, sex, evidence of cutaneous marker for Neurofibroatosis, and menopausal status were noted from the medical case files. All patients were operated by suboccipital retrosigmoid approach.

### Histopathology and immunohistochemical methods

Formaldehyde-fixed parafiin-embedded archival vestibular schwannomas specimens from the patients were obtained from the Department of Pathology, Sanjay Gandhi Postgraduate Institute of Medical Sciences, Lucknow, India. Five micron thick sections were obtained and the standard streptavidin biotin peroxidase immunohistochemical method was used for the expression of estrogen and progesterone receptors. Estrogen receptor (Clone 1D5, Dako, USA) and progesterone receptor (PgR 636, Dako, USA) monoclonal antibodies were used for the study. Estrogen and progesterone positive breast tissue was used as positive control for estrogen and progesterone receptors. For negative control, primary antibody step was omitted while performing immunohistochemistry.

## Results

**Figure 2:**
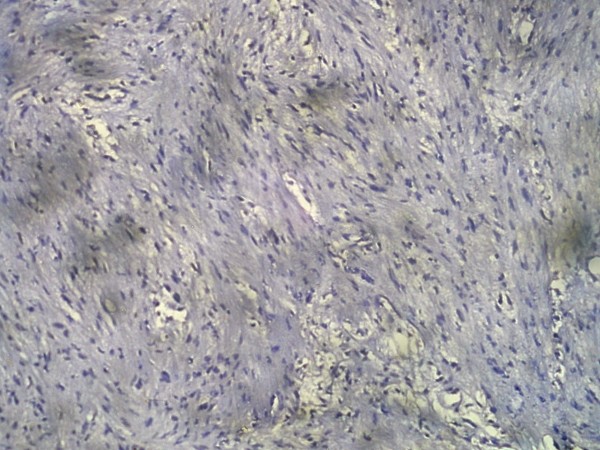
**Photomicrograph showing vestibular schwannoma with no demonstrable activity with progesterone stain**.

**Figure 3:**
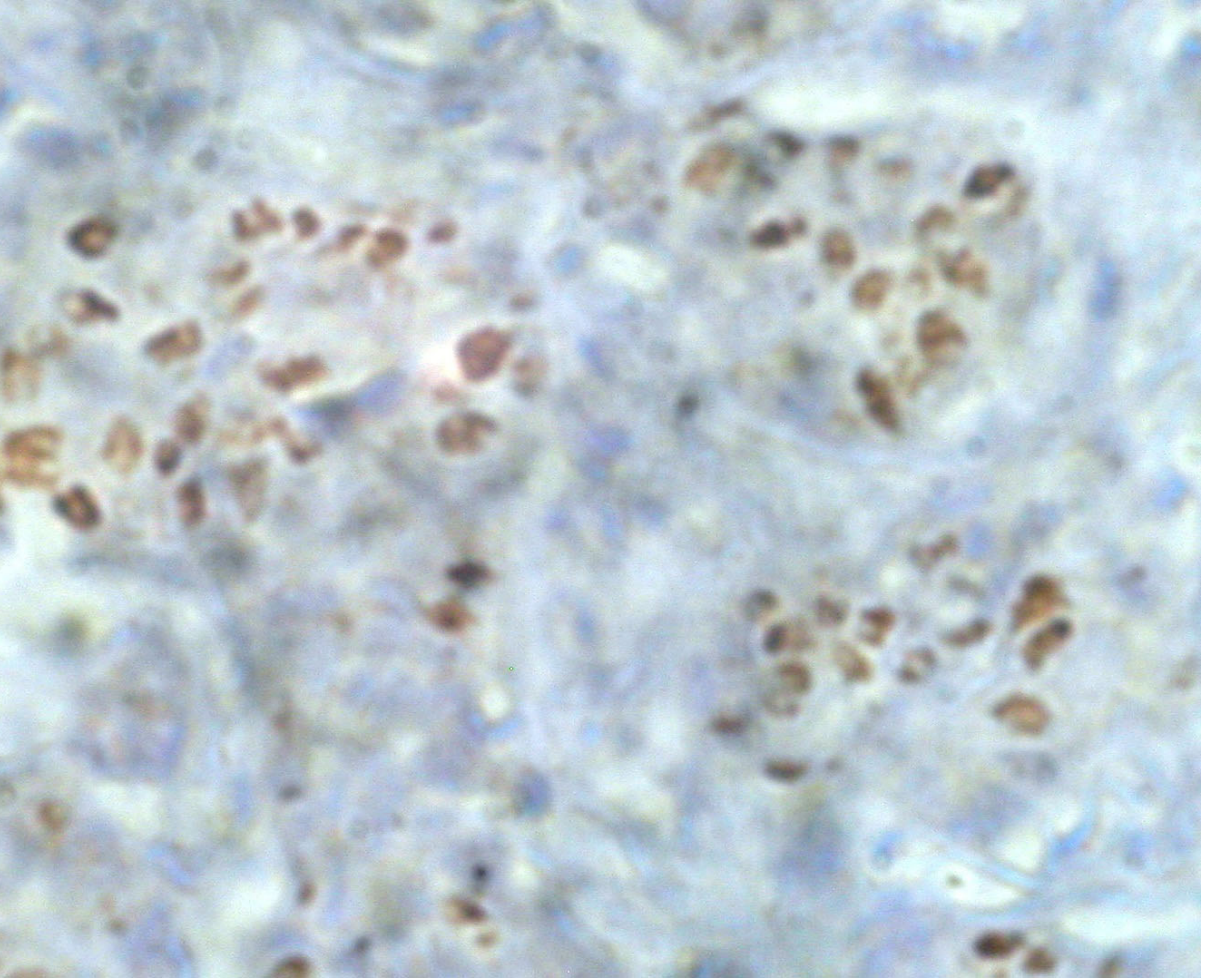
**Photomicrograph showing breast tissue with strong expression of progesterone receptors**.

Table 1 summarizes the statistical analysis of 100 cases of vestibular schwannoma included in our study. Out of 100 vestibular schwannoma cases, 63 were males and 37 were females. Age ranged from 12 years to 77 years (mean age: 37.5 years). Out of 37 females, 28 were premenopausal and 9 were postmenopausal. None was pregnant at the time of surgery. Three patients showed cutaneous markers of Neurofibromatosis. Three patients had bilateral tumors. Neither estrogen nor progesterone receptors could be detected in any of our cases by means of well known immunohistochemical method using well documented monoclonal antibodies (Figure 2). In the control specimens, a strongly positive reaction could be seen (Figure 3).

**Table 1:** Statistical analysis of 100 cases of vestibular schwannomas

**Parameters**	**Number of cases**	**Percentage**
Males	63	63

Females	37	37

Premenopausal females	28	28

Postmenopausal females	9	9

Pregnant females	0	0

Bilateral vestibular schwannomas	3	3

Presence of cutaneous markers of Neurofibromatosis	3	3

## Discussion

Vestibular schwannoma is a benign cerebellopontine angle tumor and it expresses various hormone receptors for examples estrogen, progesterone, androgen, somatostatin and glucocorticoid. The clinical significance of these receptors is that they play a possible role in tumor growth [7,14]. Studies on the expression of estrogen and progesterone receptors in vestibular schwannomas have seldom been reported. Moreover, conflicting results about the expression of estrogen and progesterone receptors in vestibular schwannomas have been reported in the literature. The level of expression has ranged from all tumor specimens being negative to 100% being positive for progesterone receptor. For estrogen receptor the level of expression ranged from all tumors being negative to 44% being positive [2-12]. Table 2 summarizes the details of these studies. These discrepancies are probably due to the divergent methodologies which have been used by various authors ranging from ligand binding studies and immunohistochemical methods to molecular techniques like polymerase chain reaction and Northern blot analysis. Positivity of estrogen and progesterone receptors in vestibular schwannomas has clinical significance as antiestrogen and/or antiprogesterone therapy may be considered as an adjunct treatment modality in vestibular schwannomas particularly in cases having small residual tumor following surgical excision.

**Table 2:** Summary of reported series of expression of estrogen and progesterone receptors in vestibular schwannomas

**Sl no**.	**Author**	**Year**	**No. of cases**	**ER positivity (%)**	**PR positivity (%)**
1	Kasantikul et al	1981	20	20	60

2	Markwalder et al	1986	16	44	Not studied

3	Monsell and Wiet	1990	37	19	17

4	Siglock et al	1990	19	100	52

5	Klinken et al	1990	18	none	none

6	Curley et al	1990	14	none	none

7	Beatty et al	1995	24	25	50

8	Carroll et al	1997	21	none	33

9	Labit-Bouvier et al	2000	69	none	10

10	Cafer et al	2008	59	none	100

11	Dalgorf et al	2008	9	none	none

12	Jaiswal et al	2009	100	none	none

Kasantikul and Brown [6] analyzed a series of 103 vestibular shwannomas and found that tumor occurred with greater frequency in women (58%), and that of 88 medium, large, and giant tumors, 64% occurred in women. Of 15 small tumors, 73% occurred in men. Moreover, tumors in women were more vascular. They performed a quanlitative immunofluorescence histochemical staining to detect estrogen-binding activity and found 1 of 8 tumors had estrogen-binding activity and the intensity of the staining was found to be much greater in the tumors from the 5 women. They concluded that estrogen may promote the growth of acoustic schwannomas by inducing proliferation of vascular endothelium, with a resultant increase in tumor vascularity. Monsell and Wiet [3] studied 37 cases of vestibular schwannomas for estrogen and progesterone receptors by radioimmunoassay and found that 19% of cases were positive for estrogen receptor, 17% cases were positive for progesterone receptors and 8% cases were positive for both the receptors. There was no correlation of estrogen receptor positivity with the sex of the patient. These results indicate that estrogen or progesterone receptor binding activity or both are present in a small subset of vestibular schwannomas. Evidence is lacking, however, that binding of estrogen to the receptor results in the growth changes in the tumor. Siglock et al [10] performed quantitative assays for estrogen, progesterone and testosterone receptors in 19 cases (10 male, 9 females) of vestibular schwannomas and found that 3 of 10 men and 7 of 9 women tumors were positive for progesterone receptors. All tumor specimens were positive for estrogen or testosterone receptors. He concluded there is need for further investigations of endocrinologic therapy as a possible treatment of acoustic neuroma. Cafer et al [11] analyzed presence of Ki-67, and estrogen and progesterone hormone receptors as well as their clinical correlates in 59 cases of vestibular schwannomas. On immunohistochemistry, all samples were positive for progesterone receptor and negative for estrogen receptor staining. Ki-67 staining was encountered in 34 of 59 (57.6%) patients, and Ki-67 values ranged from 0 per cent to 10.9 per cent (mean 1.36 per cent). There was no correlation between Ki-67, gender, tumour size and symptoms of the patients (p > 0.05). The authors concluded that oestrogen is not an important hormone in acoustic neuroma due to the absence of oestrogen receptor expression in the tissue samples. Since the progesterone receptor was expressed in all acoustic neuroma samples, they advocated further studies to find out about the inhibitory effect of antiprogesterone treatment on acoustic neuroma growth, which may be important particularly in elderly people or high-risk patients. Although Ki-67 was expressed in the majority of acoustic neuromas, it was not found an important marker in clinical practice due to a lack of any correlation with the clinical parameters. Carroll et al [6] studied expression of androgen, progesterone glucocorticoid and estrogen receptor messenger ribonucleic acid levels (mRNA) in 21 cases of vestibular schwannomas by either Northern blot analysis or the polymerase chain reaction (PCR) and demonstrated that glucocorticoid receptor mRNA was expressed in 100% of the cases. Only two male specimens were positive for androgen receptor mRNA expression by PCR-Southern blot analysis. Thirty-three percent of the schwannomas (7/21) showed a strong band for progesterone receptor mRNA by PCR-Southern blot analysis; there were an equal number of males and females in this group. Estrogen receptor mRNA levels were undetectable in all tumors examined by PCR-Southern blot analysis. They suggested that the pattern of steroid receptor expression is different in schwannomas than in meningiomas. Individual vestibular schwannoma needs to be examined for their steroid receptor mRNA expression to know whether they are responsive. Beatty et al [5] studied 24 cases of vestibular schwannomas using immunohistochemical staining and noted that estrogen receptors were positive in 6 cases and progesterone receptors were positive in 11 cases. Labit-Bouvier et al [12] analyzed 69 cases of vestibular schwannomas by immunohistochemical methods and found that 7 out of 69 were positive for estrogen receptors and none were positive for estrogen receptor.

Curley et al [8] studied 14 cases (8 males, 6 females) of vestibular schwannoma for expression of estrogen and progesterone receptors. No unequivocal positive result was noted in his study. Klinken et al [4] used immunohistochemical method and did not find estrogen and progesterone receptor positivity in any of his 18 cases (7 male, 11 females) of vestibular schwannomas. In a recent study by Dalgorf et al [13], 9 females with vestibular schwannoma were studied for expression of estrogen, progesterone and vascular endothelial growth factor (VEGF) by immunohistochemical studies using monoclonal mouse antibodies and their result for estrogen and progesterone receptor was unequivocally negative in all the nine cases while VEGF was positive in eight out of nine cases. Supporting these three studies, our study also did not demonstrate estrogen and progesterone receptors positivity in any of 100 vestibular schwannomas cases although the number of cases in our study was much higher than these studies.

## Conclusion

This study demonstrated no evidence to support the clinical hypothesis that vestibular schwannomas might be hormone dependent tumours. The methods of assay used were both specific and sensitive. No estrogen and progesterone receptor could be found in any of our 100 cases of vestibular schwannoma. Hence our study does not support a causative role of estrogen and progesterone in the growth of vestibular schwannoma as well as hormonal manipulation in the treatment of this tumor.

## Competing interests

The authors declare that they have no competing interests.

## Authors' contributions

SJ: Principal investigator - did the study, collected data and wrote the paper; VA: Co-investigator - helped in doing the study and collecting the data; AKJ: Co-investigator - collected the articles; RP: Co-investigator - provided the advice and coordinated the study; AKM: Co-investigator - gave the idea and advice for the study.
